# Experiences and Perceived Origins of Compassionate Ageism Among Older Adults During the COVID-19 Pandemic

**DOI:** 10.1093/geroni/igab046.3389

**Published:** 2021-12-17

**Authors:** Catherine Ju, Meghan McDarby, Matthew Picchiello, Brian Carpenter

**Affiliations:** 1 Washington University St. Louis, St. Louis, Missouri, United States; 2 Washington University, St. Louis, Missouri, United States; 3 Washington University in St. Louis, Olivette, Missouri, United States; 4 Washington University in St. Louis, Saint Louis, Missouri, United States

## Abstract

During the COVID-19 pandemic, there was a rise in media messages (MMs) and interpersonal behaviors (IBs) that could have been considered as reflecting compassionate ageism (i.e., ageism that stems from perceptions of older adults [OAs] as warm but incompetent). However, it is unclear how OAs experienced these MMs and IBs during the pandemic. The current study examined how OAs perceived pandemic-related MMs and IBs. We recruited 74 community-dwelling OAs (Mage = 73.18, 58% female). Participants completed a survey in which they reported the extent to which they had encountered five MMs and nine IBs throughout the COVID-19 pandemic. Then, participants rated whether they believed each MM and IB was motivated by care and how offended they were by it. Nearly all participants had encountered MMs about OAs’ vulnerability to COVID-19 (e.g., more likely to contract COVID-19, 97%; more likely to die from COVID-19, 97%). Furthermore, most participants experienced IBs emphasizing their vulnerability to COVID-19 (e.g., told by another person they had a higher likelihood of contracting COVID-19, 64%; someone had checked in on them unprompted, 63%). However, across MMs and IBs, most participants (59–100%) perceived them as motivated by care and concern, and a relatively small proportion (0–20%) reported being offended by them. Our findings underscore the importance of understanding nuances of ageism from the perspective of OAs themselves. Different forms of ageism (i.e., compassionate ageism, hostile ageism) rooted in certain stereotypes about older adults (i.e., high warmth-low competence) may uniquely shape the lived experiences of OAs.

